# Care of People with Post-COVID-19 Sequelae in the Scope of Primary Health Care: Scoping Review Protocol

**DOI:** 10.3390/ijerph192113987

**Published:** 2022-10-27

**Authors:** Karla Karolline Barreto Cardins, Severina Alice da Costa Uchôa, Lannuzya Veríssimo e Oliveira, Cláudia Helena Soares de Morais Freitas

**Affiliations:** 1 Public Health Departament, Federal University of Rio Grande do Norte, Natal 59056-000, Brazil; 2 School of Health, Federal University of Rio Grande do Norte, Natal 59078-970, Brazil; 3 Department of Clinical and Social Dentistry, Federal University of Paraíba, João Pessoa 58050-585, Brazil

**Keywords:** COVID-19, care, primary health care

## Abstract

The sequelae of COVID-19 disease significantly impact the quality of life of people, requiring long-term longitudinal care for recovery and rehabilitation. Primary health care is fundamental in the reception, monitoring, and multi-professional follow-up of post-COVID-19 symptoms and complications. This study proposes a scoping review protocol to identify and map the care process of monitoring and multi-professional follow-up of post-COVID-19 sequelae within the scope of primary health care worldwide. This protocol was based on the Joanna Briggs Institute Manual and guided by PRISMA-ScR. Articles, theses, dissertations, and official documents searched in several databases (MEDLINE/PubMed, Scopus, LILACS, Web of Science, Embase, and gray literature) will be included. Two independent reviewers will organize and select studies according to inclusion and exclusion criteria using the Rayyan software. The selected publications will be organized and summarized using a checklist proposed by the PRISMA-ScR. Simple descriptive statistics will analyze the quantitative data, while thematic analysis will be used for the qualitative data. The final scoping review will present the main findings, challenges, limitations, and potential research gaps related to the care of people with post-COVID-19 sequelae.

## 1. Introduction

The severe acute respiratory syndrome coronavirus 2 (SARS-CoV-2) was initially identified in Wuhan, China, after an outbreak of pneumonia of unidentified origin in December 2019 [1]. The virus quickly spread to all continents, leading the World Health Organization (WHO) to declare a pandemic on 11 March 2020 [2]. More than 600 million cases and 6 million deaths were already confirmed worldwide until August 2022. It presented with high prevalence in several countries of the world, such as the USA (more than 94 million infections and 1 million deaths), France (more than 34 million cases and 150,000 deaths), and Brazil (more than 34 million cases and 600,000 deaths [3]).

SARS-CoV-2 is easily spread via airborne particles, droplets, and contact of the mucosa with particles on surfaces. After infection, the clinical manifestations of the disease can vary from mild (e.g., flu-like syndrome with the presence of fever, cough, sore throat, runny nose, and headache) to severe symptoms (e.g., severe acute respiratory syndrome causing dyspnea, hypoxemia, tachypnea, and hypotension); the latter can be fatal [1,4]. About 80% of COVID-19 cases are classified as mild or moderate, and recovery may be achieved without complications. The remaining 20% develop dyspnea and hypoxemia due to pneumonia [5], requiring hospitalization in intensive care units with high mortality risk and, in most cases, needing invasive mechanical ventilation. A multicenter study conducted in Madrid with COVID-19 survivors showed that during the hospital stay, up to 88% of patients required invasive mechanical ventilation, and up to 53.7% needed a tracheostomy. These factors lead to complications in terms of quality of life, such as difficulties in mobility and in the performance of their daily life activities [6].

In this context, a secondary crisis related to post-intensive care syndrome and disease sequelae may take place for months or years after hospitalization, with symptoms such as fatigue, dyspnea, chest tightness, cognitive deficits, and psychological effects [7], impairing quality of life, activities of daily living, the return to work [8], and physical, cognitive, and emotional health. This is a challenge since there are no evidence-based international guidelines on how to manage the long-term sequelae left by the disease, requiring studies on the long-term effects on morbidity to plan an effective healthcare delivery and capacity [9,10].

Long-term care is needed, therefore, to promote the recovery and rehabilitation of COVID-19 sequelae and the impairments related to hospitalization, optimize physical and cognitive functioning, and reduce the risk of disabilities and morbidities [11], and the recommendations after hospital discharge are less known, requiring better knowledge about these individuals during the long-term recovery, would help clinicians and decision-makers optimize these patients’ management in the community. In this way, appropriate rehabilitation programs have been recommended, as they can improve mobility issues and maximize the functional return of COVID-19 survivors [5].

Moreover, actions and services in the health care network, coordinated by the Primary Health Care (PHC), must be developed to address the demand from the user and family reception to the monitoring and multi-professional follow-up of post-COVID-19 symptoms and complications [4]. Thus, Starfield (2002) developed an approach to characterize PHC, defining its essential attributes, which are: first contact access, longitudinality, comprehensiveness, and the coordination of care. The first attribute emphasizes the need for primary care to be a gateway to the health system, ensuring the provision of accessible services. Longitudinality implies the responsibility for regular care over time with the same professionals. Integrality suggests the provision of all services and levels of health care, considering the organic, psychological, and social needs of individuals. Additionally, the coordination of care involves vertical and horizontal actions, integration between services and professionals to plan care, define flows, monitor treatment plans, and solve less frequent and more complex problems, contributing to comprehensiveness and timely resolution [12].

In this way, the PHC needs to manage most COVID-19 cases and the consequences left by the pandemic since this disease has changed how primary care works. In this context, PHC must frequently change and adapt to the new demands of the population since the COVID-19 pandemic caused immediate and profound social, economic, and health impacts [13]. This scenario will demand the reinvention of PHC and the planning of assistance strategies for the care and monitoring of post-COVID-19 cases [11].

Studies on post-COVID-19 rehabilitation are still limited, and searching PubMed and other databases, we found some articles such as “Long COVID and Health Inequities: The Role of Primary Care” [14] and “Post-COVID-19 syndrome: A call for continuity of multidisciplinary care” [15]; however, a preliminary search conducted in electronic databases (i.e., Joanna Briggs Institute [JBI] Evidence Synthesis, The Cochrane Library, PROSPERO, and Medline) in September 2022 found no reviews or study protocols on the topic. Therefore, believing that this scoping review can support care practices within the scope of PHC, the objective is to identify and map the care process of monitoring and multi-professional follow-up of post-COVID-19 sequelae within the scope of PHC worldwide. We believe that this study might identify gaps in the current literature and support the practice of PHC health professionals.

## 2. Materials and Methods

Scoping reviews use the scientific literature to (1) identify the types of evidence available in a field of knowledge, (2) clarify the main concepts existing in the literature, (3) examine how research is conducted in a particular field, (4) identify the main characteristics or factors related to a concept, (5) precede a systematic review, and (6) identify and analyze existing knowledge gaps [16].

This scoping review protocol was registered in the Open Science Framework (10.17605/OSF.IO/DYABR) and performed according to the JBI manual [17]. The research question and relevant publications were identified, the studies were selected, the data were extracted, and the evidence was analyzed and synthesized to build this protocol [18,19].

### 2.1. Review Question

The Population, Concept, Context (PCC) mnemonic was used to formulate the research question, which provides information to help to identify possible knowledge gaps, present key concepts, quantify aspects of interest, and explain the practices and evidence of a specific topic [20]. Thus, the review question will be, “how is the care of people with post-COVID-19 sequelae conducted within the scope of PHC?”

P—people with post-COVID-19 sequelae;

C—care;

C—PHC in the world.

The care concept within the PHC is longitudinal since it is related to the reception, monitoring, and resolution of health problems based on the attributes of the PHC. It is also emphasized by teamwork, bonding with users, and supporting the management sector, favoring people with post-COVID-19 symptoms and complications [21,22]. In this way, the review will contribute to a better understanding of these aspects of care in PHC.

### 2.2. Inclusion and Exclusion Criteria

We will include studies (e.g., articles, theses, dissertations, or official documents) that investigated the care process of monitoring and multi-professional follow-up of post-COVID-19 sequelae within the scope of PHC. Studies related to care provided at other levels of specialized and hospital care not coordinated by PHC will be excluded in addition to duplicate publications, reviews, editorials, theoretical essays, expert opinions, and abstracts of events.

### 2.3. Search Strategies

As recommended in all types of JBI reviews, a three-step search strategy will be utilized to reach the greatest number of publications and gray literature. Each step is indicated in this section of the protocol.

#### 2.3.1. Identification of Descriptors and Keywords

A survey of keywords, descriptors, and synonyms in health sciences included in the titles, abstracts, and indexed terms of publications related to the topic was conducted using the Medical Subject Headings (English) and the Descriptors in Health Sciences (Portuguese) (Table 1).

#### 2.3.2. Definition of Databases

The definition of a high-sensitivity search strategy was carried out with the help of a librarian (Table 2), and after that, data collection will be conducted in the BVS/LILACS, MEDLINE/PubMed, Scopus, Web of Science, and EMBASE databases. Searches will also be performed in the following gray literature: Google Scholar, Digital Library of Theses—CAPES, Open-Access Theses and Dissertations, and ProQuest Global Dissertations & Theses.

Two researchers will conduct the searches using the vocabulary found and the Boolean operators AND and OR. The Boolean operator OR will be used between words of the same group, while the AND operator will be used between a set of words of different groups. The search in the databases will be performed in November 2022, and the language of the studies will not be limited; the consolidation and presentation of the results will be undertaken in English, and only publications from 2019 onward will be included.

#### 2.3.3. Search for Additional Sources in the References of Publications

The references of publications included in the review will also be analyzed for eligible studies. The corresponding authors will be contacted via email for additional information if necessary.

### 2.4. Search for Studies

Data collection will be adapted for each database. Rayyan software (Qatar Foundation, Doha, Qatar) [23] will be used to facilitate data collection and duplicate removal. Two reviewers will use a random sample of 25 studies in a pilot test to verify the inclusion criteria and a minimum agreement of 75% between the reviewers.

In the study, two blinded reviewers will individually evaluate the titles and abstracts of all of the identified studies using Rayyan software [23]. Disagreements between the reviewers will be debated for consensus, and another reviewer will be consulted if needed.

### 2.5. Study Selection

The study selection will be detailed in a flow diagram following the steps proposed by the Preferred Reporting Items for Systematic Review and Meta-Analyses: (1) identification, (2) selection, (3) eligibility, and (4) inclusion [24]. In this step, the reviewers will perform a new search in all of the databases to verify if new studies can be included.

### 2.6. Data Extraction and Coding

The data will be extracted and included if aligned with the objective and study question of the scoping review. Two independent reviewers will extract these data to reduce the chances of errors and biases using a data extraction form developed in Microsoft Excel^®^, based on the JBI model and adapted by the authors (Table 3). The form may be updated during the survey to deepen the understanding of the topic [17].

### 2.7. Analysis

Simple descriptive statistics (absolute and relative frequencies) will be conducted using the SPSS software (IBM Corp, Armonk, NY, USA, version 24). Qualitative data analysis (thematic analysis) will be performed to identify meanings and patterns to answer the research question. The type of study and level of evidence of the study design will also be analyzed.

### 2.8. Reporting of Results

This step will be divided into (1) data analysis, (2) exposure of results linked to research questions, and (3) interpretation of the implications of results for other research and services [19]. As outcomes of interest, we expect to find information based on the attributes of the PHC, mainly longitudinality, comprehensiveness, and the coordination of care, in search of actions and strategies that are being developed by the team, discussing whether PHC has fulfilled its role to guarantee health care for people with post-COVID-19 sequelae. All of the results will be discussed based on the relevant literature, and the PRISMA-ScR will guide the final report, and evidence synthesis will be described using tables, diagrams, and thematic maps for better visualization of results. A meta-analysis of the information will also be conducted in case of studies that can be combined. A narrative summary will follow the mapped data and report the relationships between the results and the review objective and question.

The summarized results will be presented to stakeholders and researchers to improve the review discussion and provide socialization and knowledge transfer. The latter will share preliminary results, discuss strategies for disseminating results, favor knowledge exchange, and encourage the search for new evidence or field of research not present in the review [19].

### 2.9. Summary of Evidence, Conclusions, and Implications

A summary of the results related to the study objectives will be developed, and gaps in the knowledge area will be highlighted for future studies (e.g., systematic reviews).

## 3. Conclusions

This scoping review may contribute to scientific production and guide new research with the intention of improving practice in the health services provided to people with post-COVID-19 sequelae.

Our results will be shared with stakeholders, presented in a scientific article, and published in an open-access and peer-reviewed journal, supporting knowledge dissemination within the scientific community. The necessary changes in this protocol will be properly justified and informed in the final publication.

## Figures and Tables

**Table 1:** Descriptors used according to the Population, Concept, Context mnemonic.

Mnemonic	DeCS	Keywords	MeSH	Keywords
P (Population)	COVID-19	Post-COVID-19 syndrome Long COVID Post-COVID-19 sequelae	COVID-19	COVID-19
C (Concept)	Care	Health Care Periodic Care Assistance	Care	Health Care
C (Context)	Primary Health Care	Primary Health Care First Level of Assistance	Primary Health Care	Primary Health Care

**Table 2:** Search strategy based on LILACS, MEDLINE/PubMed, Scopus, Web of Science, and EMBASE databases.

Source of Information	Search Strategy
BVS/LILACS	“COVID-19/CO” or “COVID-19/RH” [Descritor de assunto] or “pós-COVID” [Palavras]—117 “COVID-19/CO” or “COVID-19/RH” [Descritor de assunto] or “pós-COVID” [Palavras] and “atencao primaria a saude” [Descritor de assunto]—1
MEDLINE/PubMed	(“post-acute COVID-19 syndrome” [Supplementary Concept] OR “COVID-19/complications” [MeSH Terms] OR “COVID-19/rehabilitation” [MeSH Terms]) AND (primary health care [MeSH Terms])—179
Scopus	TITLE-ABS-KEY (“post-acute COVID-19 syndrome” OR “COVID-19/complications” OR “COVID-19/rehabilitation”)—617 (TITLE-ABS-KEY (“post-acute COVID-19 syndrome” OR “COVID-19/complications” OR “COVID-19/rehabilitation”) AND TITLE-ABS-KEY (“primary health care”)—3
Web of Science	(“post-acute COVID-19 syndrome” OR “COVID-19/complications” OR “COVID-19/rehabilitation”)—544 (“post-acute COVID-19 syndrome” OR “COVID-19/complications” OR “COVID-19/rehabilitation”) and (“primary health care”)—1
EMBASE	(“post-acute COVID-19 syndrome” OR “COVID-19/complications” OR “COVID-19/rehabilitation”)—694 (“post-acute COVID-19 syndrome” OR “COVID-19/complications” OR “COVID-19/rehabilitation”) and (“primary health care”)—1

**Table 3:** Data extraction form.

Variable	Description
Publication title	Publication title
Type of material	If article, dissertation, thesis, or official documents
Year of publication	Year of publication
Context of publication	Location where the study was conducted (country)
Academic background of the author	Academic background of the first author
Objective	Objective of the study
Types of studies	If randomized or non-randomized controlled trials, cohort, case–control, cross-sectional, descriptive observational, ecological, or qualitative studies
Post-COVID-19 sequelae	Highlight the post-COVID-19 sequelae described in the study
Results	Highlight the main results of the study
Challenges and limitations	Highlight possible challenges or limitations of the study

## Data Availability

Not applicable.
